# Fat embolism syndrome: Clinical and imaging considerations: Case report and review of literature

**DOI:** 10.4103/0972-5229.40948

**Published:** 2008

**Authors:** Nissar Shaikh, Ashok Parchani, Venkatraman Bhat, Marie Anne Kattren

**Affiliations:** **From:** Department Anesthesia/ICU and Pain Management, Hamad Medical Corporation, Doha-Qatar; 1 Department of Radiology, Hamad Medical Corporation, Doha-Qatar

**Keywords:** Fat embolism syndrome, imaging, magnetic resonance imaging

## Abstract

Fat embolism syndrome (FES) is a serious clinical disorder occurring after trauma, orthopedic procedures and rarely in non-traumatic patients. Fat emboli develop in nearly all patients with bone fractures, but they are usually asymptomatic. Small number of patients develop signs and symptoms of various organ system dysfunction due to either mechanical obstruction of capillaries by fat emboli or due to hydrolysis of fat to fatty acids. A triad of lung, brain and skin involvement develop after an asymptomatic period of 24 to 72 hours. This symptom complex is called FES. The incidence reported is up to 30%, but many mild cases may recover unnoticed. Diagnosis of fat embolism is clinical with nonspecific, insensitive diagnostic test results. Treatment of fat embolism syndrome remains supportive and in most cases can be prevented by early fixation of large bone factures. Here we report two cases of traumatic fat embolism, which were diagnosed initially by Gurd's criteria and subsequently confirmed by typical appearance on magnetic resonance imaging (MRI) of the brain in these patients. These patients were successfully treated with supportive management. In conclusion, diagnosis of FES needs high index of suspicion, exclusion of other conditions and use of clinical criteria in combination with imaging. Magnetic resonance imaging of the brain is of great importance in diagnosis and management of these patients.

## Introduction

Fat embolism syndrome was first described in 1873 by Von Bergman[1] in patients with fracture of the femur. Whereas two decades earlier than this description of fat embolism syndrome, fat emboli were noted by Zenker[2] in a crush injury patient.

Fat embolism develop in nearly all patients with long bone fractures or during orthopedic surgical procedures but are usually asymptomatic. However, in a small number of cases, patients develop signs and symptoms due to multisystem dysfunction, mainly involving the lungs, brain and skin. For these cases the term fat embolism FES is applied.[3] Exact incidence of fat embolism syndrome is not known, but Fabian *et al.* reported an incidence of up to 30% in their study.[4] The diagnosis of fat embolism remains a difficult task as there are no universal criteria for diagnosis and laboratory studies are non-specific.

Here we present two cases of fat embolism syndrome suspected initially on clinical grounds subsequently correlated with findings on magnetic resonance imaging of the brain.

Diagnosis is much easier when imaging observations are correlated clinically.

## Case 1

A 21-year-old dark male patient, involved in a road traffic accident, was admitted to the hospital with a closed fracture of the femur and tibia. On admission he was fully awake, obeying commands and hemodynamically stable.

After 12 h of admission he became tachypnoeic, developed tachycardia and started to desaturate. He was put on oxygen supplementation via facemask, which improved his saturation. He remained stable for the next 12 h but later became irritable, confused, was not obeying commands and started to desaturate again.

Patient was intubated, sedated and shifted to ICU and connected to a ventilator. He was put on Midazolam and Remifentail infusions. Blood workup showed slight drop in his hemoglobin and platelets levels.

X-ray chest on admission showed diffuse bilateral infiltrates; ECG showed right ventricular strain pattern with tachycardia. Accordingly as per Gurds classification he was diagnosed as a case of Fat embolism syndrome.

Computed tomographic (CT) study of the head was done next day showed a low attenuation area in the occipital region. CT examination of the chest revealed bilateral basal airspace filling lesions. MRI of the brain done on day three, in T2 weighted images, showed multiple, bilateral, scattered, hyperintense lesions in the deep pariventricular region, centrum semiovale, corpus callosum and bilateral basal ganglionic regions. Few tiny similar bright spots were noted in the pons and cerebellar peduncles as well. Clinical features and MRI appearances were suggestive of cerebral fat embolism and/or associated hypoxic sequelae. With supportive treatment in ICU, hydration and enteral feeding he started to improve, was arousable and started to obey commands. On day 11 he was operated for (fixation of) fracture femur and subsequently was extubated on day 13.

He remained in ICU for a further five days and was shifted from nasogastric feedings to oral diet, transferred on day 18 to the ward fully awake, obeying commands, was hemodynamically stable and was sent home after three days.

## Case 2

A Twenty-two years old male pedestrian was involved in a road traffic accident and sustained closed fracture of the femur on the right side. He was a known case of homocysteinurea. He was fully awake and conscious at the time of admission. There were no other injuries; He had normal vital signs and was admitted to the orthopedic ward.

On the third day following admission he became tachypnoeic, tachycardic, irritable and started to desaturate and was put on supplemental oxygen. He continued to deteriorate; showed decreased level of consciousness hence was intubated and was shifted to ICU put on ventilatory support, started on remifentanil and midazolam infusions. He was found having petichea all over his upper trunk including axillae. Fundoscopy showed retinal hemorrhages. Blood workup showed drop in platelet counts and hemoglobin level, with increased coagulation profile. He received blood and blood products accordingly. Septic work-up done for his persistent fever was found to be negative.

X-ray chest showed diffuse scattered infiltrates. He was diagnosed as fat embolism syndrome by Gurd's criteria.

**Figure 1:**
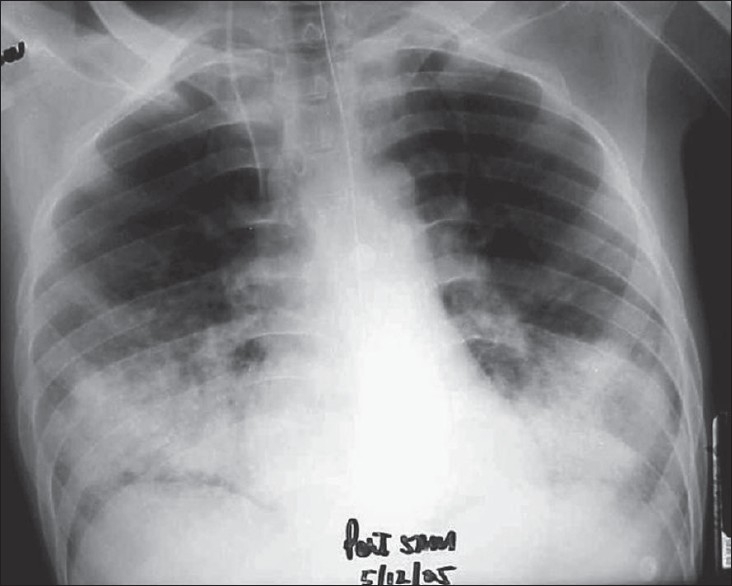
AP radiograph of chest showing bilateral basal air space filling lesions consolidation

**Figure 2:**
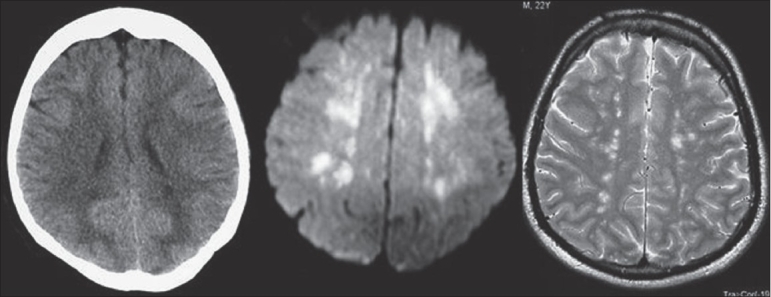
CT image showing minimal hypodense changes in periventricular region, which are more evident in DWI and T2WI as areas of high signals. Constellation of findings along with clinical data is characteristic for FES

CT examination of head showed subtle hypodense changes in centrum semiovale and periventricular region and CT chest showed bilateral basal consolidation [Figure 1]. MRI was done on the sixth day of admission, showed multiple hyperintense areas in the cerebral white matter and basal ganglia, in diffusion weighted images (DWI) and T2 weighted images (T2WI), consistent with the impression of Fat Embolism [Figure 2]. He started to improve after good hydration, and good intensive care management and other supportive measures. He had fixation of the femur done on day15 and subsequently improved further and was extubated on day 19.

He remained in the ICU for two more days. He became fully awake, started to obey commands, was started on normal diet and was transferred to the ward on the 21^st^ day with stable vitals signs and was subsequently discharged from the hospital after two days.

## Discussion

Fat embolism could be difficult to diagnose. Fat embolism occurs in closed fractures of large bones and pelvis, but rarely it can occur with nontraumatic conditions such as pancreatitis, sickle cell disease, liposuction, decompression sickness and fatty liver.[5]

Actual incidence of fat embolism is rather unknown as mild cases of fat embolism may be unnoticed. Fat embolism typically manifests 24 to 72 h after initial insult. Affected patients presents with the classic triad of hypoxemia, neurological abnormality and petechial rash. Pathology of FES is poorly understood. According to Glosslin *et al.*,[6] FES results when large fat droplets are released into venous system and result in physical obstruction of pulmonary system vessel. Whereas Baker *et al.*,[7] incriminates free fatty acid, local hydrolyses of triglycerides together with excessive mobilization of free fatty acid resulting in production of toxic intermediaries. Delay of 24-72 h after insult indicates production of toxic intermediaries. Among the clinical triad pulmonary manifestation are the earliest to appear. These include tachypnoea, dyspnoea and cyanosis which progress to respiratory failure in 10% of the cases. Cerebral changes are seen in 86% of cases and these range from headache and confusion, to stupor, rigidity, convulsions and coma.

Petechial rash is reddish brown, non palpable, appears on the upper chest, neck and conjunctiva. It results from occlusion of dermal capillaries and increased capillary fragility.[10] The distribution is related to fat particles floating in the aortic arch, like oil in water and embolized to non dependent skin areas via aortic arch vessels (subclavian or carotid arteries.)[10] Other signs are not specific.

For diagnosis of fat embolism there is no universal criteria, various criteria were proposed by different authors.

According to Gurd's *et al.*,[8] diagnosis of FES need at least two major criteria or one major and four minor criteria to be present in order to diagnose FES [Table 1].

**Table 1:** Gurd's criteria[8]

Major criteria	Petechial rash
	Respiratory insufficiency
	Cerebral involvement
Minor criteria	Fever
	Retinal changes
	Jaundice
	Renal signs
	Thrombocytopenia
	Anaemia
	High ESR
	Fat macroglobinneamia

Schonfeld *et al.*,[11] gave quantitative means of diagnosis of FES [Table 2] Cumulative score > 5 are required for diagnosis Lindegue *et al.*,[12] suggested that FES can be diagnosed on basis of respiratory status alone [Table 3].

**Table 2:** Schonfeld criteria[11]

Criteria	Score
Petechiae	5
X-ray chest diffuse infiltrâtes	4
Hypoxemia	3
Fever	1
Tachycardia	1
Tachycardia	1
Confusion	1

**Table 3:** Lindegue criteria[12]

1 Sustained po2 < 8 kpa
2 Sustained pco2 > 7.3 kpa.
3 Sustained respiratory rate > 35/min, inspite of sedation
4 Increased work of breathing, dyspnoea, tachycardia, anxiety

For rapid and specific diagnosis of FES few author suggested brochoalveolar lavage[13] but invasive nature of the procedure limits the usefulness of this technique.

Imaging studies are the most useful in diagnosis of FES, X-ray chest radiograph often show increased pulmonary markings and flake like pulmonary shadow (snowstorm appearance).[12]

Computed tomographic study is insensitive in the diagnosis of FES. It may be of some value in excluding alternative clinical considerations. MR imaging is the most sensitive technique in demonstrating cerebral changes of FES. Sensitivity of the DWI sequences[14] and fluid attenuated inversion recovery images[15] over conventional imaging, in this situation is highlighted in the recent literature. Lesions can be demonstrated as early as within 30 min of ictus in experimental studies using these sequences. The findings described in FES are multiple small, non-confluent hyperintense lesions on DWI and T2-weighted images, usually situated within the cerebral white matter and deep gray matter often at the watershed zones. Typically lesions are distributed in centrum semiovale, subcortical white matter, ganglionic regions and in thalami. The number and size of the lesions is variable but correlates with the degree of neurologic disability as measured by the GCS.[16] These findings are thought to represent micro infarcts arising from fat emboli occluding cerebral arterioles may further lead to permanent pathologic changes such as cyst formation and gliosis. Some of the lesions may represent vasogenic edema due to the toxic effects of free fatty acids and may show complete resolution. Free fatty acids have been shown to be particularly toxic to brain tissue. MRI is more sensitive than CT, now increasingly available hence the modality of choice in the investigation of FES. Takahashi *et al.* graded these changes into four grades, based on size and distribution of the lesions in T2 weighted images in to grade 0 (normal) to grade 3 (most severe). Authors even showed that resolution of high intensity MRI lesions paralleled with clinical recovery.[16]

MRI changes of FES and changes of hypoxic encephalopathy in the subacute phase, appear different. Lesion distribution in both instances involve cerebral cortex, basal ganglia, as grey matter is more vulnerable to global ischemia and anoxia than white matter. Global ischemic lesions tend to show confluent diffuse changes,[17] rather than multifocal discrete lesions of FFS. Following entities can be considered in the differential diagnosis of disseminated hyperintense lesions on T2WI and DWI: namely diffuse axonal injury, areas of vasogenic edema associated with microinfarcts, foci of gliosis, dilated perivascular Virchow-Robin spaces and demyelinating disease.[18]

In our patient group we did not find contrast enhancement useful whereas DWI and FLAIR images consistently showed higher sensitivity in lesion detection. Distribution of the lesion was classical except for one patient who had more extensive lesions which included posterior fossa and fronto-temporal lobes.

Treatment of fat embolism, is only supportive. The role of steroids is controversial. Good hydration, restoration of intravascular volume is important. Albumin has been recommended for resuscitation, as it restores blood volume, also binds with fatty acids hence may decrease the tissue injury.[19] Incidence of FES can be decreased by early stabilization of long bone fractures.

## Conclusion

Diagnosis of fat embolism syndrome may be complex. High index of suspicion in an appropriate clinical setting, in combination of cerebral MRI and chest imaging findings are the key for diagnosis of fat embolism syndrome. MR imaging, including diffusion weighted sequences should be the initial imaging procedure of choice when diagnosis is clinically entertained.
